# Why an Increase of TSH in Populations With Initially Mild-to-Moderate Iodine Deficiency Can Be Good News

**DOI:** 10.3389/fnut.2022.910160

**Published:** 2022-06-17

**Authors:** Thomas Remer

**Affiliations:** DONALD Study Centre Dortmund, Institute of Nutrition and Food Science (IEL), University of Bonn, Dortmund, Germany

**Keywords:** iodine deficiency, iodine status, subclinical hypothyroidism, thyrotropin, TSH, thyroid gland

Normal function of the hypothalamic-pituitary-thyroid axis implies an increase in thyrotropin (TSH) secretion if circulating thyroxin (T4) and/or triiodothyronine (T3) fall. A fall in thyroxine does occur in moderate to severe iodine deficiency when amounts of iodine taken up and stored in the gland no longer suffice to maintain adequate hormone synthesis and secretion. Accordingly, there is overwhelming consent that severely iodine-deficient populations generally have higher serum TSH concentrations than adequately supplied populations (1).

Despite that, higher circulating TSH levels are also observable along with excessive iodine intake (2–4). Correspondingly, a number of studies found U-shaped relationships for different indicators of thyroid disorders, including thyroglobulin and TSH levels over a wide range of iodine supply from clearly deficient to excessive iodine intake (5–8).

Referring to this, Laurberg et al. reported that below iodine intake levels of around 220 μg/day rather no increased risk of subclinical hypothyroidism, i.e., of relevant elevations of TSH is present for adults (6). Zimmermann et al. (5) who examined 6–12 years old children, notified that there need be no fear of increases in the prevalence of elevated TSH or thyroglobulin levels, as long as urinary iodine concentrations (UIC) are below 300 μg/Liter in this age group.

On the other hand, there is a growing number of studies reporting increases in TSH that occur at clearly lower iodine intake or urinary iodine excretion levels (both in children and adults) than those specified by the aforementioned authors. Corresponding results have been observed in the Korean National Health and Nutrition Examination Survey 2013–2015 (9). A recent large Chinese study reported raising serum TSH concentrations already from urinary iodine concentrations of 50 μg/L upward up to around 500 μg/L in a well characterized reference population, i.e., in adults without personal or family histories of thyroid dysfunction, without visible or palpable goiter, without having different kinds of thyroid-related antibodies, and without taking any medication except estrogens (10). This finding of a TSH rise along with an increase in iodine nutrition occurring already at lower initial iodine intake levels is in line with another Chinese examination in children (11) and a Danish examination in adults (12) both comparing regions of low iodine intake (median UIC < 100 μg/L, or median 24-h iodine excretion around 50 μg/day) with regions of correspondingly higher intake and excretion levels. Additional evidence comes from observations, e.g., from Italy (13) and again from Denmark (14), describing enhancing effects of iodine fortification on population's serum TSH concentrations in populations with initial mild to moderate iodine deficiency.

Accordingly, the remarkable increases in the diagnosis of subclinical hypothyroidism (i.e., the rise in the number of exceedings of given TSH cut-offs) after iodine nutrition has improved in certain regions or populations with mild to moderate iodine deficiency (15–18), may reflect—at least in part—a consequence of an iodine rise-related right shift of the respective population's TSH distribution (19). Such a TSH right shift along with an appropriately increased iodine intake has been reported recently also for thyroid-healthy children and adolescents (20). All these findings, in turn, may explain a relevant part of the current relative large number of patients treated for hypothyroidism and receiving thyroxine, although probably not requiring thyroid hormone therapy, as has to some extent been documented and discussed recently (21). In concert with this, among many non-pregnant adults treated for subclinical hypothyroidism, the use of thyroid hormone therapy has not been found to be associated with improvements in general quality of life or thyroid-related symptoms (22). As the latter authors concluded, these findings did not support the routine use of thyroid hormone therapy in adults diagnosed to have subclinical hypothyroidism, i.e., to have TSH values exceeding certain cut-offs without fT4 reductions.

As long as two decades ago, Andersen et al. (23) performed a study during which they collected urine and blood samples monthly for 1 year in healthy men living in an area of mild to moderate iodine deficiency. The authors found a positive relationship between circulating TSH and the over-the-year-averaged iodine excretion rates exceeding 50 μg/day, whereas the correlation was inverse below this excretion rate, again revealing a U-shape between iodine status and TSH. Remarkably, the nadir of the later occurred at the iodine excretion level close to the UIC range with the respective lowest TSH values of those studies that reported TSH measurements for both lower and higher iodine intakes (11, 14, 16, 20, 24). Figure 1 schematically represents the potential U-shaped relationship between iodine status and related circulating TSH levels suggesting a physiological nadir of the TSH-iodine intake relationship already when iodine supply is still insufficient.

**Figure 1:**
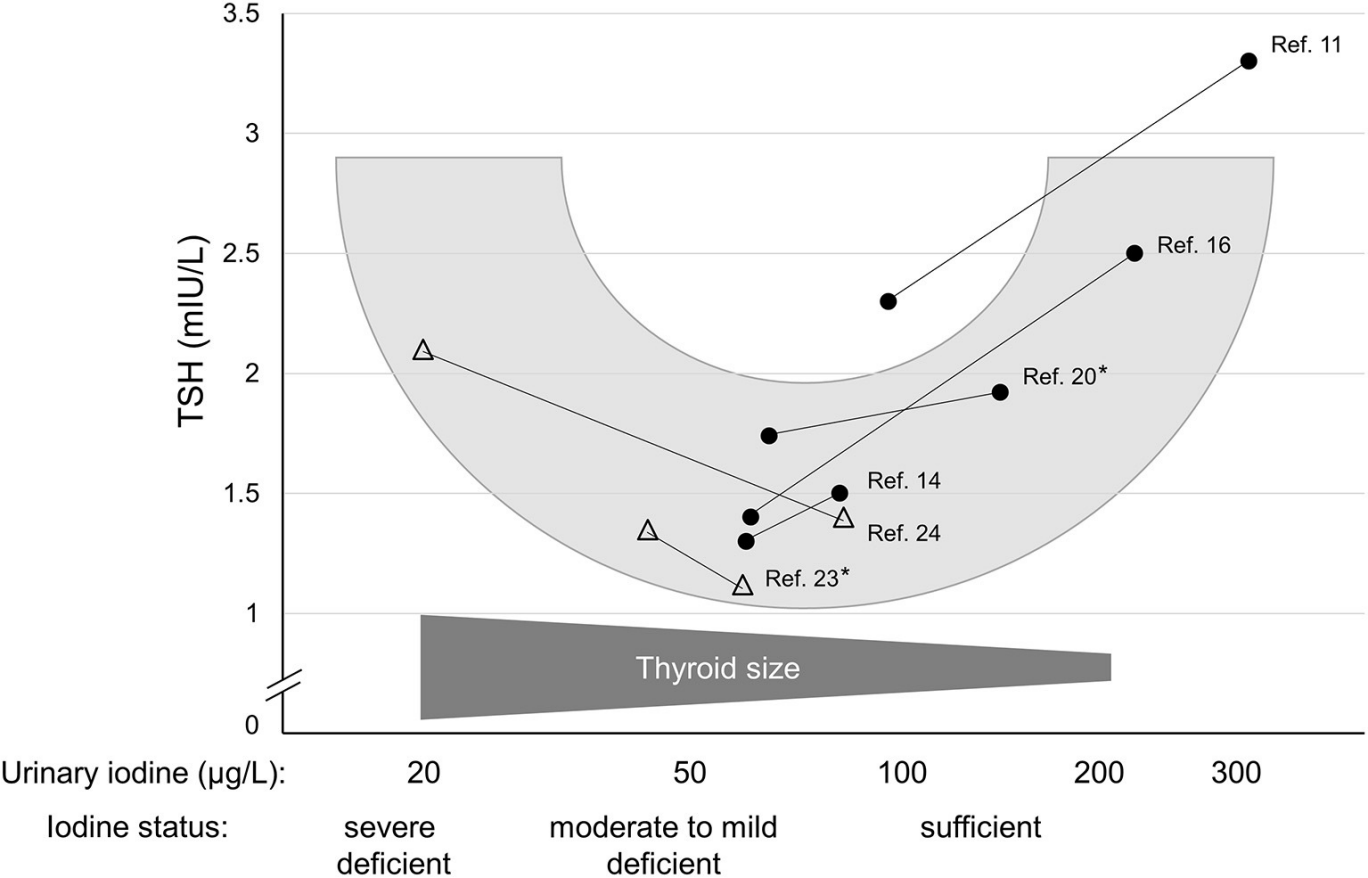
Schematic representation of the probable U-shaped relationship between circulating TSH and iodine nutritional status (urinary iodine, logarithmized). The filled circles show median urinary iodine measurements and related TSH values of studies describing a mild iodine deficiency along with a situation of an (at least somewhat) improved iodine supply. Open triangles show a moderate or an almost severe iodine deficiency each along with an improved iodine status. Asterisks denote studies that have provided iodine measurements not as μg/L, but as μg/day.

One reason why this “early” TSH nadir, rather occurring in the range of mild to moderate iodine deficiency has not been detected in a number of epidemiological studies may be that TSH assays with less analytical accuracy and precision could have been used (5, 25).

Although the underlying mechanisms for a reduced TSH signaling particularly during mild to moderate iodine deficiency are not yet definitely clarified, it can be assumed that the major parts of the TSH increases related to improvements in iodine nutrition are of physiological and not of pathophysiological nature. Correspondingly increased TSH values have not only been documented in specifically screened thyroid healthy children and adolescents (20), but also in thyroid healthy adults (10).

Improving iodine nutrition and thus iodine availability to the “mild-to-moderately iodine-deficient” thyroid gland will reduce mass and number of the gland's cells, of which each remaining cell consequently will require a higher TSH signal to maintain thyroid hormone adequacy. Apart from an inhibitory effect of an increased iodine level on TSH signaling within the thyroid cell, i.e., a lowered sensitivity of the gland to TSH (26), also the reduced capillary vascularization of the thyroid that is reducing in size (26), may contribute to a physiologically raised TSH requirement.

Taken together, whenever progress against iodine deficiency is seen, e.g., due to a successful salt iodization in a mild to moderate deficient area, a regular increase in the population's TSH levels should be expected and although definite proof is still lacking it may be interpreted as the gland's physiological response to an improved iodine availability. Unfortunately, corresponding TSH increases are commonly interpreted—and in a way medically “mis-termed”—as increases in the prevalence of subclinical hypothyroidism, which may have contributed to the probable over-prescription of thyroxine, recently reported (21).

## Author Contributions

The author confirms being the sole contributor of this work and has approved it for publication.

## Conflict of Interest

The author declares that the research was conducted in the absence of any commercial or financial relationships that could be construed as a potential conflict of interest.

## Publisher's Note

All claims expressed in this article are solely those of the authors and do not necessarily represent those of their affiliated organizations, or those of the publisher, the editors and the reviewers. Any product that may be evaluated in this article, or claim that may be made by its manufacturer, is not guaranteed or endorsed by the publisher.
